# Different obesity indicators and their correlation with hypertension, diabetes, and dyslipidemia in 35–74 years rural residents in Northwest China

**DOI:** 10.3389/fendo.2025.1346193

**Published:** 2025-06-09

**Authors:** Ting Yin, Jing Wang, XueQing Lan, Jiaxing Zhang, Qingan Wang, Jiangwei Qiu, Tao Ma, Yi Zhao, Yuhong Zhang

**Affiliations:** ^1^ Department of Epidemiology and Health Statistics, School of Public Health, Ningxia Medical University, Yinchuan, Ningxia, China; ^2^ Key Laboratory of Environmental Factors and Chronic Disease Control, Ningxia Medical University, Yinchuan, Ningxia, China; ^3^ General Hospital of Ningxia Medical University, Public Health School, Ningxia Medical University, Yinchuan, Ningxia, China; ^4^ Peking University First Hospital Ningxia Women and Children's Hospital (Ningxia Hui Autonomous Region Maternal and Child Health Hospital), Ningxia, China; ^5^ Department of Nutrition and Food Hygiene, School of Public Health, Ningxia Medical University, Yinchuan, Ningxia, China

**Keywords:** obesity indicators, hypertension, diabetes, dyslipidemia, rural residents

## Abstract

**Objective:**

This study aimed to explore the cut-off value of 10 obesity indicators, including BF% (Body Fat Ratio, BF%), BMI (Body Mass Index, BMI), WHR (Waist-to-Hip Ratio, WHR), WHtR (Waist-to-Height Ratio, WHtR), BAI (Body Adiposity Index, BAI), OBD (Obesity Degree, OBD), CI (Conicity Index,CI), AVI (Abdominal Volume Index, AVI), ABSI (A Body Shape Index, ABSI) and BRI (Body Roundness Index, BRI), and investigate their relationship between different anthropometric indices of obesity indicators and their correlation to hypertension, diabetes, and dyslipidemia in rural residents aged 35–74 years in Ningxia, an autonomous region of northwest China.

**Methods:**

The study participants were interviewed by questionnaire (including demographic characteristics such as age, education status, economic status, and lifestyle variables such as exercise frequency, smoke, alcohol, tea, spice, and vinegar consumption), bio-impedance body composition analysis, and blood laboratory test. The t-test and chi-square test were used to compare the characteristics of different groups, and the receiver operating characteristic curve was used to analyze the correlation of different indicators and explore their cut-off values.

**Results:**

The study comprised 14,926 participants, of whom 39.80% (5948/14,926) were male, and the mean age of the study population was 56.75 ± 9.74 years. The waist circumference had the greatest influence on obesity indicators, and BMI, AVI, and BRI are most susceptible to anthropometric indicators. WHtR had the largest AUC (Area Under the ROC Curves, AUC) for predicting obesity in both male and female. In addition, we provided a recommended cut-off value of BMI, WHR, WHtR, BAI, OBD, CI, AVI, ABSI and BRI. WHtR had the largest AUC for predicting diabetes, hypertension, and dyslipidemia, while WHtR served as a good predictive indicator (all P<0.001).

**Conclusion:**

Waist circumference is closely related to obesity. Therefore, there is a great significance to carry out long-term health management education among the population, change the unhealthy lifestyle and promote the metabolic health for the primary prevention of cardiovascular diseases.

## Graphical Abstract

Different obesity indicators and the calculation.

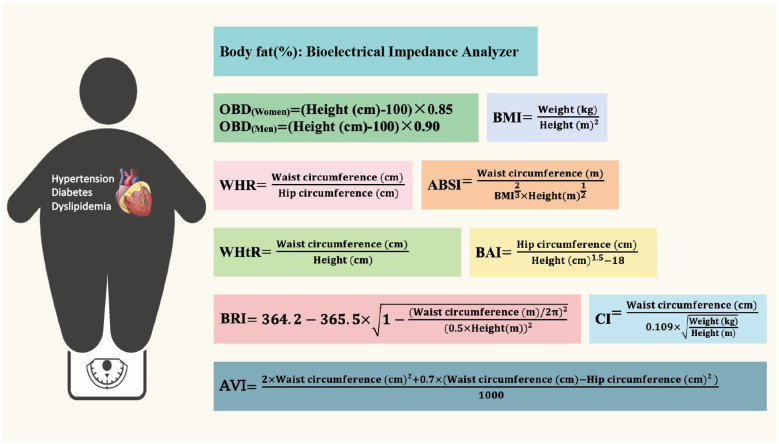

## 1 Introduction

Obesity is a metabolic disease caused by the body’s calorie intake exceeding consumption, resulting in the accumulation of body fat or excessive weight growth (1). Obesity affects people’s quality of life and is closely related to many cardiovascular diseases. Especially, among a series of social problems brought about by population aging, the increasing number of middle-aged and elderly people with obesity has gradually become a public health problem that cannot be ignored (2, 3). According to the Chinese Center for Disease Control and Prevention, more than 8.1% of Chinese adults were obese in 2018, three times as many as in 2004 (4). The Report on Nutrition and Chronic Diseases of Chinese Residents (2020) showed that the overweight and obesity rate of urban and rural residents of all age groups continues to rise, with more than half of adult residents being overweight or obese (5). A cross-sectional study of 1,577,094 Chinese adult participants showed that 14.1% were obese according to BMI (Body Mass Index, BMI). Men were higher than women (standardized prevalence of obesity was 17.6% and 9.6%, respectively). Compared with participants with normal BMI, obese individuals had a higher prevalence of assessed complications, primarily prediabetes, dyslipidemia, and hypertension, and higher BMI brings more complications (6).

Glucose and lipid metabolism are the basis of life activities and play an important role in maintaining human health (7–9). Abnormal glucose and lipid metabolism is a major risk factor leading to the occurrence and death of cardiovascular diseases worldwide (10, 11). Hypertension is the most common chronic noncommunicable disease in China and the most important risk factor for cardiovascular diseases, which seriously consumes medical and social resources (12). Studies have found that the proportion of abnormal blood pressure in middle-aged people is increasing, and the prevalence rate of obesity-related hypertension in Chinese adults aged 45 and above is 22.7%. However, the awareness rate and treatment compliance are lacking, especially in rural areas (13). Study shows that the prevalence of diabetes in adults is on the rise (14). The incidence of diabetes in the elderly in China is about 30.0%, which is much higher than the average adult level, and seriously affects the quality of life and survival of the elderly (15). The epidemiological survey on dyslipidemia in China shows that the prevalence of dyslipidemia varies greatly due to different groups, regions and cultural characteristics, and the prevalence of dyslipidemia is higher in obese people (16).

Currently, standard measures that define obesity varies widely, body fat percentage (BF%) is the gold standard for diagnosing obesity (17). In addition to the body mass index (BMI) and waist-hip ratio (WHR), Waist-to-height ratio (WHtR), waist circumference (WC), neck circumference (NC) are used to evaluate obese, leading to inconsistencies in result (18, 19). At the same time, these indexes could not distinguish subcutaneous and visceral fat. The cut-off values of WC and WHR are also affected by gender. Therefore, new compound obesity indicators was proposed. Research shows that Body adiposity index (BAI) can better reflect the weight loss of patients with mild Obesity (20). The Obesity degree (OBD) calculates the proportion of the actual body mass exceeding the ideal body mass (21). Conicity index (CI) reflects the accumulation of abdominal fat (22), and Abdominal volume index (AVI) is a comprehensive index based on traditional measurements (such as waist circumference and hip circumference), which is mainly used to assess the accumulation of visceral fat (23). A body shape index (ABSI) adds waist circumference to BMI, which can be used to infer the risk of death from obesity-related diseases (24). The Body roundness index (BRI) evaluates the distribution of body fat by calculating the ratio of waist circumference to height. Studies have shown that it can be used as an anthropometric index to evaluate dyslipidemia (25). However, among these traditional obesity indicators and new obesity indicators, which is better associated with hypertension, diabetes and dyslipidemia, and its cut-off value needs to be further studied in Chinese population.

Therefore, this study chose BF% as the gold standard of obesity indicators, to explore the consistency of other standards, and investigate the cut-off value of each indicator in Ningxia rural adults and their relationship with obesity-related diseases, such as hypertension, diabetes, and dyslipidemia.

## 2 Materials and methods

### 2.1 Study population

Data for this study was obtained from a population-based cohort study conducted at Ningxia Medical University between March 2018 and May 2019, comprising a random sample of more than 14,926 men and women aged 35 to 74 years in Pingluo County, Shizuishan City and Qingtongxia City, Wuzhong City in Ningxia.

### 2.2 Inclusion and exclusion criteria

Inclusion criteria were (i) male and female adults aged between 35 and 74 (born between 1943 and 1982), (ii) registered permanent residents in the selected investigation sites (those who stay at home for more than five months throughout the year), (iii) no serious physical disability and are able to communicate normally; (iv) registration report of morbidity and death of the disease belongs to the administrator of the local health department. Exclusion criteria were: (i) participants who planned to leave the local area for various reasons within the past year and could not be followed up, (ii) have been seriously ill or hospitalized in the last two weeks, (iii) pregnant or breastfeeding women.

The sample size was calculated using n=400*(q/p). Previous research results showed that the prevalence rate of diabetes among adults in rural areas of Ningxia was 4.21%, and the sample size for this study was about 9,102. According to the inclusion and exclusion criteria, 14,926 participants were finally included in the study.

**Figure 1:**
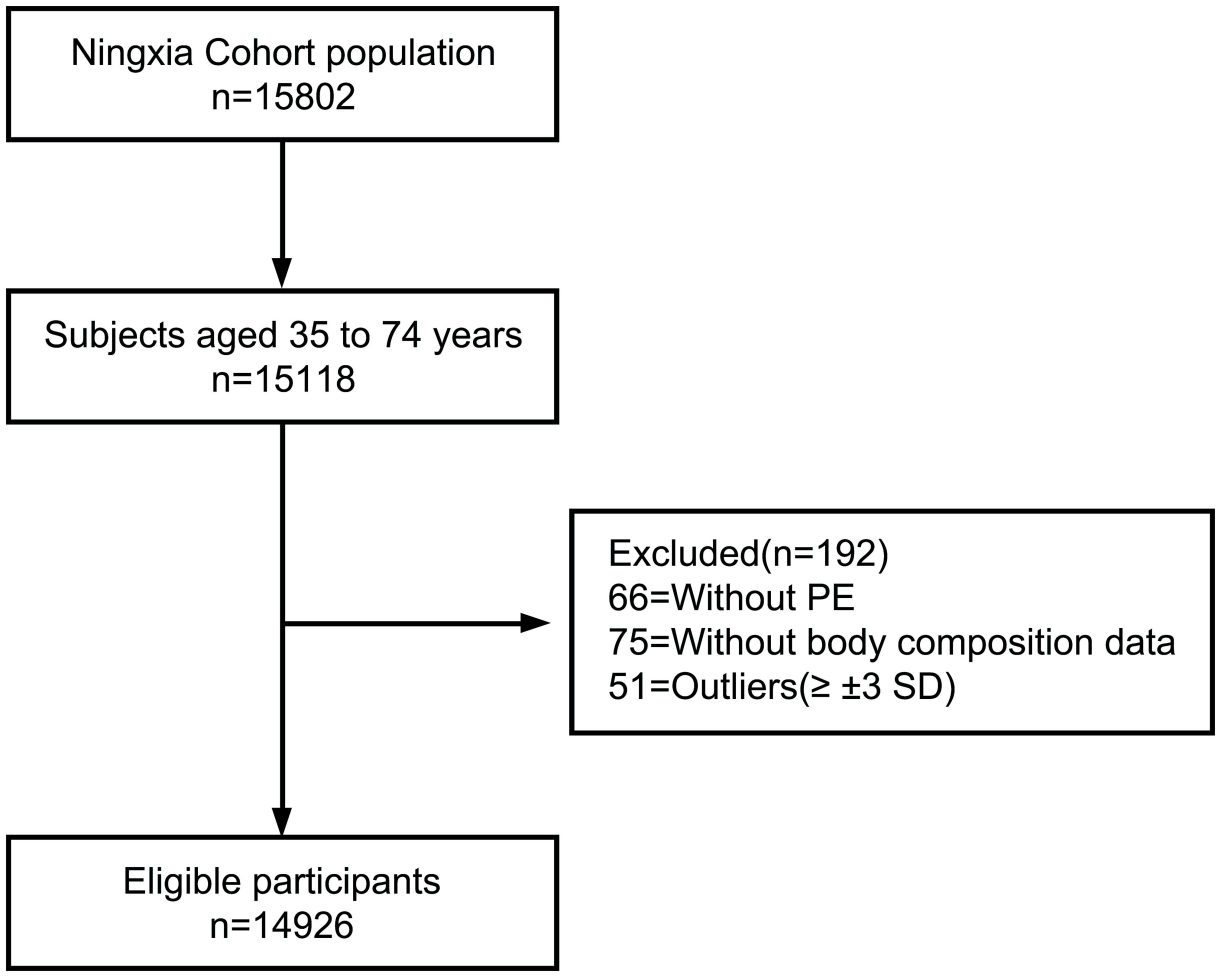
Respondent screening process for the participants.

All participants met the above four inclusion criteria to formally participate in the study and provided written informed consent. All investigations were performed in accordance with the Declaration of Helsinki and approved by the Ethical Committee of Ningxia Medical University. The specific process is shown in **Figure 1**.

### 2.3 Data collection

All participants underwent a questionnaire interview to collect demographic data, followed by a physical examination for anthropometric variables. After blood samples were sent to the clinical laboratory testing department, biochemical measurements were performed according to standard laboratory procedures. All operations were performed by trained investigators and processed by trained and experienced laboratory technicians.

The studies involving human participants were reviewed and approved by the Ethics Committee of Ningxia Medical University (No. 2018-021). All participants provided their written informed consent to participate in this study.

### 2.4 Variables

#### 2.4.1 Demographic data

After providing informed consent, all participants were interviewed by questionnaire to obtain demographic data, such as age, sex, marital status, education status, economic status, and certain lifestyle variables, including exercise frequency, whether they smoked or not, and their alcohol, tea, spice, and vinegar consumption. Each participant was given a study identification (ID) number to ensure prevention bias.

#### 2.4.2 Anthropometric variables

The anthropometric variables were measured by well-trained investigators. Body height (cm) was measured, in light clothing and without shoes, with a height bar. Brachial artery blood pressure and pulse were measured using an electronic sphygmomanometer. Each measurement was repeated twice; if the measured values were within 0.5 cm of one another, their average was calculated. If the difference between the two measurements exceeded 0.5 cm, then a third measurement was conducted. Weight, BMI, waist circumference, neck circumference, and other anthropometric variables were acquired using a bioelectrical impedance analyzer (BIA; Inbody Co., Seoul, Korea), according to manufacturer guidelines. The BIA calculates the resistance of body tissues to an electrical signal sent through the hands and feet. Participants removed extra clothes, such as shoes, coats, sweaters, and metal accessories, such as earrings, rings, and watches, and stood on a balance scale with bare feet and grasped the handles of the BIA. The examination took approximately 30 s.

#### 2.4.3 Biochemical variables

Blood samples were obtained between 8:00 and 10:00 am, following overnight fasting, and were used to perform biochemical analyses by means of standard laboratory enzymatic methods. These analyses included fasting blood glucose (FBG, mmol/L), total cholesterol (TC, mmol/L), triglyceride (TG, mmol/L), high-density lipoprotein cholesterol (HDL-C, mmol/L), and low-density lipoprotein cholesterol (LDL-C, mmol/L) levels. The serum was centrifuged, aliquoted, and stored at -80°C.

### 2.5 Diagnostic standards

#### 2.5.1 Definitions of obesity

Ten definitions of obesity were compared: (i) Body fat ratio: Derived from the results of the bioelectrical impedance analyzer. (ii) The WHO definition of BMI (kg/m^2^), $\text{BMI}=\frac{Weight\;\left(kg\right)}{Height\;{\left(m\right)}^{2}}$ , BMI greater than 25.0 were considered obese for Asians. (iii) $\text{WHR}=\frac{Waist\;circumference\;\left(cm\right)}{Hip\;circumference\;\left(cm\right)}$ , if the Waist-to-hip ratio (WHR) greater than 0.8 in women or 0.9 in men, it was defined as central obesity. (iv) $\text{WHtR}=\frac{Waist\;circumference\;\left(cm\right)}{Height\;\left(cm\right)}$ , Waist-to-height ratio (WHtR), WHtR greater than 0.5 was identified as a cut-off value for obesity in this study. (v) $\text{BAI}=\frac{Hip\;circumference\;\left(cm\right)}{Height\;{\left(cm\right)}^{1.5}-18}$ , Body adiposity index (BAI). (vi) ${\text{OBD}}_{\left(\text{Women}\right)}=\left(Height\;\left(cm\right)-100\right)\times 0.85$ , ${\text{OBD}}_{\left(\text{Men}\right)}=\left(Height\;\left(cm\right)-100\right)\times 0.9$ , Obesity degree (OBD), participants who exceeded 20% of the standard weight were diagnosed as obese. (vii) Conicity index (CI), $\text{CI}=\frac{Waist\;circumference\;\left(cm\right)}{0.109\times \sqrt{\frac{Weight\;\left(kg\right)}{Height\;\left(m\right)}}}$ . (viii) Abdominal volume index (AVI), 


$$\text{A}\text{V}\text{I}=\frac{2\times Waist\;circumference\;{\left(cm\right)}^{2}+0.7\times \left(Waist\;circumference\;\left(cm\right)-Hip\;circumference\;{\left(cm\right)}^{2}\right)}{1000}.$$

(ix) A body shape index (ABSI), $\text{ABSI}=\frac{Waist\;circumference\;\left(m\right)}{BMI^{\frac{2}{3}}\times Height\;{\left(m\right)}^{\frac{1}{2}}}$ . (x) Body roundness index (BRI), $\text{BRI}=364.2-365.5\times \sqrt{1-\frac{{\left(Waist\;circumference\;\left(m\right)/2\pi \right)}^{2}}{{\left(0.5\times Height\;\left(m\right)\right)}^{2}}}$ . As shown in **Graphical Abstract**.

#### 2.5.2 Definitions of hypertension, diabetes, and dyslipidemia

The diagnosis of hypertension was based on the criteria recommended by Guidelines for hypertension management in the elderly in China (2023) (26), defined as systolic blood pressure ≥140 mmHg and diastolic blood pressure ≥90 mmHg. According to China National Guidelines for Prevention and Treatment of Diabetes Mellitus (2022) (27), FPG ≥7.0 mmol/L was considered as diabetes mellitus. The diagnosis for dyslipidemia refers to Chinese Lipid Management Guidelines (2023) (28), patients with hypercholesterolemia (TC≥5.20 mmol/L), hypertriglyceridemia (TG≥1.70 mmol/L), or low-high-density lipoprotein cholesterolemia (HDL-C< 1.00 mmol/L) were diagnosed with dyslipidemia.

### 2.6 Statistical analyses

Absolute number (percentage, %) and mean ± standard deviation (SD) were used describe the categorical data. Chi-Square test for categorical data and t-test for continuous data were used to compare the difference between different groups. Pearson correlation was used for correlation analysis. Receiver operating characteristic (ROC) analyses were then used to calculate the area under the ROC curves (AUC) between dyslipidemia and anthropometric measures, adjusted for age and sex. All analyses were performed using SPSS statistical software version 26.0. All tests were 2-tailed, and P< 0.05 was considered statistically significant.

## 3 Results

### 3.1 Demographic and clinical characteristics of the study participants

Totally 15,802 participants were enrolled, after the exclusion of individuals younger than 35 years or older than 74 years, and missing data on physical examination questionnaire (including age, Marital status, level of education, et al.) and body composition data (including weight (kg), height (m), SBP (mmHg), et al.), 14,926 participants were included finally. The basic characteristics of the study population are shown in **Figure 1**.

Among the participants, 39.80% (5948/14,926) were male, the mean age of the study population was 56.75 ± 9.74 years, most of them were married (93.4%) and only had a primary school education (67.3%), and females showed less smoking and alcohol consumption, and more tea and vinegar consumption, compared to men. Males tended to have higher anthropometric measures, such as the neck circumference, waist circumference, arm and thigh circumference (all P<0.001). For the obesity indicators, BMI showed no significant difference between males and females in this study, and WHR showed little statistical difference (P=0.015). BF%, WHtR, BAI, and CI were higher in females than males, OBD, AVI, and BRI were higher in males than females, and ABSI was almost the same both groups (all P<0.05). Regarding biochemical status, males tended to have higher blood pressure and FBG levels, females has higher TC, HDL-C, and LDL-C levels (all P<0.001), and TG showed no statistically significant difference between the two groups (**Table 1**).

**Table 1:** Basic characteristics of study subjects.

Variables	Total (n=14,926)	Males (n=5948)	Females (n=8978)	*P*-value
Demographic characteristics				
Age (years)	56.75 ± 9.74	58.59 ± 9.59	55.54 ± 9.69	<0.001
Marital status, n (%)				
Married	13,946 (93.4)	5682 (95.5)	8264 (92.0)	<0.001
Divorce/Widowed	938 (6.3)	232 (3.9)	706 (7.9)	
Unmarried	42 (0.3)	34 (0.6)	8 (0.1)	
Education, n (%)				
Primary school and below	10,038 (67.3)	3616 (60.7)	6422 (71.5)	<0.001
Junior high school	4323 (29.0)	2005 (34.0)	2318 (25.8)	
Senior high school and above	565 (3.7)	327 (5.3)	238 (2.7)	
Economic status				
(RMB/year), n (%) Less than 20,000	7927 (53.1)	3169 (53.3)	4758 (53.0)	0.349
20,000~50,000	5286 (35.4)	2074 (34.9)	3212 (35.8)	
More than 50,000	1713 (11.5)	705 (11.8)	1008 (11.2)	
Exercise(days/week),				
n (%)< 3	9925 (66.5)	3903 (65.6)	6022 (67.1)	0.153
3-5	1549 (10.4)	624 (10.5)	925 (10.3)	
6-7	3452 (23.1)	1421 (23.9)	2031 (22.6)	
Current Smoking (yes), n (%)	2204 (14.8)	2090 (35.1)	114(1.3)	<0.001
Alcohol consumption (yes), n (%)	3598 (24.1)	2314 (38.9)	1284 (14.3)	<0.001
Tea consumption (yes), n (%)	9007 (60.3)	4169 (70.1)	4838 (53.9)	<0.001
Spice consumption (yes), n (%)	9860 (66.1)	3896 (65.5)	5964 (66.4)	0.241
Vinegar consumption (yes), n (%)	11,317 (75.8)	4586 (77.1)	6731 (75.0)	0.003
Anthropometric variables				
SBP (mmHg)	135.37 ± 19.51	136.01 ± 19.28	134.95 ± 19.65	<0.001
DBP (mmHg)	83.26 ± 12.62	84.40 ± 12.99	82.50 ± 12.31	<0.001
Weight (kg)	64.01 ± 10.56	69.00 ± 10.61	60.70 ± 9.12	<0.001
Height (m)	1.60 ± 0.07	1.66 ± 0.06	1.56 ± 0.06	<0.001
NC (cm)	36.89 ± 3.04	38.43 ± 2.74	35.87 ± 2.79	<0.001
CC (cm)	93.52 ± 6.89	96.84 ± 6.66	91.31 ± 6.11	<0.001
WC (cm)	87.07 ± 9.76	88.64 ± 10.46	86.03 ± 9.11	<0.001
HC (cm)	94.40 ± 5.29	95.91 ± 5.25	93.40 ± 5.07	<0.001
LAC (cm)	31.40 ± 3.04	32.14 ± 3.09	30.91 ± 2.91	<0.001
RAC (cm)	31.38 ± 3.10	32.10 ± 3.22	30.91 ± 2.92	<0.001
LTC (cm)	48.98 ± 3.17	49.98 ± 3.18	48.31 ± 2.98	<0.001
RTC (cm)	49.30 ± 3.39	50.39 ± 3.40	48.58 ± 3.19	<0.001
Obesity indicators				
BF% (%)	31.74 ± 7.59	27.01 ± 6.57	34.87 ± 6.52	<0.001
BMI (kg/m^2^)	24.94 ± 3.40	24.96 ± 3.39	24.93 ± 3.40	0.698
WHR	0.92 ± 0.06	0.92 ± 0.07	0.92 ± 0.06	0.015
WHtR	0.54 ± 0.06	0.53 ± 0.06	0.55 ± 0.06	<0.001
BAI	28.77 ± 3.62	26.85 ± 3.07	30.04 ± 3.39	<0.001
OBD	52.36 ± 7.93	59.58 ± 5.74	47.58 ± 5.05	<0.001
CI	1.26 ± 0.07	1.26 ± 0.07	1.27 ± 0.06	0.001
AVI	15.41 ± 3.45	16.00 ± 3.76	15.03 ± 3.16	<0.001
ABSI	0.08 ± 0.00	0.08 ± 0.00	0.08 ± 0.00	<0.001
BRI	161.07 ± 21.77	170.28 ± 22.70	154.97 ± 18.79	<0.001
Biochemical variables				
FBG (mmol/L)	5.65 ± 1.66	5.72 ± 1.74	5.60 ± 1.61	<0.001
TG (mmol/L)	1.70 ± 1.16	1.69 ± 1.23	1.71 ± 1.11	0.231
TC (mmol/L)	4.86 ± 1.05	4.71 ± 1.09	4.96 ± 1.01	<0.001
HDL-C (mmol/L)	1.35 ± 0.35	1.29 ± 0.36	1.40 ± 0.34	<0.001
LDL-C (mmol/L)	2.83 ± 0.85	2.76 ± 0.93	2.88 ± 0.80	<0.001

*P-value from two independent samples; t-test for all continuous variables and chi-square test for categorical variables.

(SBP, Systolic Blood Pressure; DBP, Diastolic Blood Pressure; NC, Neck Circumference; CC, Chest circumference; WC, Waist Circumference; HC, Hip Circumference; LAC, Left Arm Circumference; RAC, Right Arm Circumference; LTC, Left Thigh Circumference; RTC, Right Thigh Circumference; FBG, fasting blood glucose; FBG, Fasting Blood Glucose; TC, Total Cholesterol; TG, Triglyceride; HDL-C, High-Density Lipoprotein Cholesterol; LDL-C, Low-Density Lipoprotein Cholesterol; BMI, Body Mass Index ), WHR (Waist-to-Hip Ratio, WHR), WHtR (Waist-to-Height Ratio, WHtR), BAI (Body Adiposity Index, BAI), OBD (Obesity Degree, OBD), CI (Conicity Index,CI), AVI (Abdominal Volume Index, AVI), ABSI (A Body Shape Index, ABSI) and BRI (Body Roundness Index, BRI.).

### 3.2 Correlation between obesity indicators and anthropometric and biochemical variables

Pearson’s correlation coefficients were used to measure the correlation between obesity indicators and anthropometric and biochemical variables (**Table 2**). Anthropometric measures, such as waist circumference, left and right arm circumference, were significantly correlated with obesity indicators (all P<0.001). Among them, BMI was affected by nine anthropometric measures in addition to height, most closely associated with hip circumference, left and right arm circumference. AVI and BRI were also affected by seven measures in addition to height and thigh circumference, AVI most influenced by waist circumference, left and right arm circumference, while BRI associate closely to weight, waist circumference and chest circumference. Meanwhile, waist circumference had the most influence on obesity assessment indicators, including BMI, WHR, WHtR, CI, AVI and BRI, height correlated best with OBD. Biochemical variables showed lower correlation coefficients with obesity indicators, only blood pressure may had little effect.

**Table 2:** Correlation between obesity indicators and anthropometric and biochemical variables.

Variables	BF%	BMI (kg/m^2^)	WHR	WHtR	BAI	OBD	CI	AVI	ABSI	BRI
Weight (kg)	0.274	**0.796**	0.663	0.645	0.150	0.562	0.580	**0.883**	0.285	**0.950**
Height (m)	-0.479	-0.046	0.153	-0.187	-0.722	**0.989**	0.132	0.258	0.183	0.582
NC (cm)	0.257	**0.778**	0.710	0.687	0.208	0.463	0.629	**0.860**	0.355	**0.884**
CC (cm)	0.273	**0.836**	0.713	0.722	0.257	0.455	0.628	**0.895**	0.322	**0.915**
WC (cm)	0.592	**0.879**	**0.917**	**0.900**	0.387	0.248	**0.866**	**0.996**	0.590	**0.934**
HC (cm)	0.444	**0.918**	0.616	0.748	0.417	0.326	0.525	**0.872**	0.154	**0.853**
LAC (cm)	0.517	**0.936**	**0.763**	**0.868**	0.504	0.194	0.684	**0.925**	0.338	**0.846**
RAC (cm)	0.504	**0.914**	0.741	**0.846**	0.493	0.188	0.664	**0.900**	0.325	**0.822**
LTC (cm)	0.309	**0.801**	0.401	0.562	0.323	0.382	0.318	0.716	-0.038	0.746
RTC (cm)	0.307	**0.806**	0.392	0.561	0.333	0.374	0.306	0.713	-0.055	0.739
SBP (mmHg)	0.200	0.227	0.239	0.244	0.147	-0.013^†^	0.204	0.223	0.130	0.183
DBP (mmHg)	0.162	0.271	0.252	0.258	0.095	0.081	0.258	0.282	0.188	0.267
FBG (mmol/L)	0.032	0.068	0.050	0.043	-0.001^†^	0.035	0.064	0.063	0.055	0.065
TG (mmol/L)	0.157	0.202	0.214	0.201	0.127	0.001^†^	0.197	0.206	0.137	0.178
TC (mmol/L)	0.212	0.158	0.179	0.147	0.153	-0.122	0.167	0.120	0.141	0.061
HDL-C (mmol/L)	0.029^*^	-0.057	-0.044	-0.044	0.017^*^	-0.115	-0.035	-0.086	0.001^†^	-0.111
LDL-C (mmol/L)	0.114	0.086	0.093	0.087	0.068	-0.042	0.083	0.072	0.070	0.049

^†^P-value >0.05 and shows no significant statistical difference. ^*^P-value<0.05, and others all significant at the<0.001 level.

Measures with highest correlation coefficients for each variable in bold.

(SBP, Systolic Blood Pressure; DBP, Diastolic Blood Pressure; NC, Neck Circumference; CC, Chest circumference; WC, Waist Circumference; HC, Hip Circumference; LAC, Left Arm.

Circumference; RAC, Right Arm Circumference; LTC, Left Thigh Circumference; RTC, Right Thigh Circumference; FBG, fasting blood glucose; FBG, Fasting Blood Glucose; TC, Total Cholesterol; TG, Triglyceride; HDL-C, High-Density Lipoprotein Cholesterol; LDL-C, Low-Density Lipoprotein Cholesterol).

### 3.3 Cut-off points between different obesity indicators in population

The Area Under the ROC Curves (AUC) between different obesity indicators after adjusting for sex are shown in **Table 3**. The objective was to investigate the predictive value of alternative obesity indicators, and determine the optimal cut-off value that best balanced sensitivity and specificity, relative to BF%. This study shows that WHtR had the largest AUC for predicting obesity in both sexes, the cut-off value is 0.52 for males and 0.55 for females. Followed by BMI and AVI, the cut-off value is 24.35 and 14.87 for males, 24.85 and 14.79 for females. In the whole population, the sensitivity and specificity of BMI and WHtR cut-off estimation were better, AVI was more suitable for males. OBD showed no significant diagnostic value. Men have higher values for BAI, OBD, AVI, and BRI, and lower values for BMI, WHR, and WHtR, compared to females, ABSI has the same as the cut-off point of 0.08 in both sexes.

**Table 3:** Adjusted AUC and cut-off points, sensitivity, and specificity between different obesity indicators in population.

Variables	Total (n=14926)				Male (n=5948)				Female (n=8978)			
	AUC (95% CI)	Cut-off	Sensitivity (%)	Specificity (%)	AUC (95% CI)	Cut-off	Sensitivity (%)	Specificity (%)	AUC (95% CI)	Cut-off	Sensitivity (%)	Specificity (%)
BMI	**0.893(0.888, 0.899)**	**24.35**	**81.93**	**78.70**	**0.901(0.894, 0.909)**	**24.35**	**79.45**	**83.57**	**0.907(0.901, 0.914)**	**24.85**	**78.06**	**82.71**
WHR	**0.873(0.867, 0.879)**	**0.92**	**75.88**	**78.67**	**0.892(0.884, 0.900)**	**0.92**	**75.35**	**85.66**	**0.873(0.865, 0.881)**	**0.93**	**69.64**	**81.89**
WHtR	**0.947(0.944, 0.950)**	**0.54**	**79.17**	**85.30**	**0.947(0.942, 0.952)**	**0.52**	**83.26**	**89.69**	**0.945(0.941, 0.950)**	**0.55**	**85.17**	**83.99**
BAI	**0.859(0.853, 0.866)**	**28.64**	**66.86**	**73.45**	**0.852(0.843, 0.862)**	**26.70**	**71.00**	**82.88**	**0.864(0.855, 0.872)**	**29.58**	**78.19**	**75.66**
OBD	0.357(0.348, 0.367)	53.99	40.99	60.17	0.415(0.400, 0.430)	60.98	35.76	50.77	0.394(0.380, 0.408)	199.00	45.82	38.56
CI	**0.859(0.853, 0.865)**	**1.26**	**72.40**	**77.96**	**0.879(0.871, 0.888)**	**1.25**	**75.13**	**83.47**	0.847(0.838, 0.855)	1.26	73.89	73.98
AVI	**0.875(0.870, 0.881)**	**14.79**	**79.49**	**81.02**	**0.904(0.896, 0.911)**	**14.87**	**81.02**	**82.03**	**0.898(0.891, 0.905)**	**14.79**	**77.74**	**80.97**
ABSI	0.721(0.712, 0.730)	0.08	53.83	74.49	0.769(0.757, 0.781)	0.08	69.40	70.44	0.676(0.663, 0.689)	0.08	53.01	70.50
BRI	0.758(0.750, 0.767)	159.37	70.26	75.82	0.831(0.821, 0.841)	163.10	77.95	70.28	0.814(0.804, 0.824)	155.73	67.97	75.15

Anthropometric measures with the highest AUC value in bold.

(BMI, Body Mass Index; WHR, Waist-to-Hip Ratio; WHtR, Waist-to-Height Ratio; BAI, Body Adiposity Index; OBD, Obesity Degree; CI, Conicity Index; AVI, Abdominal Volume Index; ABSI, A Body Shape Index; BRI, Body Roundness Index).

### 3.3 Diagnostic value of different indicators in hypertension, diabetes and dyslipidemia

The AUC for hypertension, diabetes, and dyslipidemia and the different obesity indicators are shown in **Table 4**. Overall, the accuracy of each obesity indicator in predicting hypertension, diabetes, and dyslipidemia was not very high (all AUC<0.7). WHtR and BMI had the same AUC for hypertension (0.606, 95%CI: 0.595, 0.617) and diabetes (0.573, 95%CI: 0.552, 0.594) while WHtR also had high predictive level for dyslipidemia (0.625, 95%CI: 0.616, 0.635) (all P<0.001). The AUC of other obesity evaluation indicators, including BAI, OBD, CI, AVI, ABSI and BRI, were all around 0.5, suggesting low predictive value.

**Table 4:** Adjusted AUC of different obesity indicators evaluating hypertension, diabetes, and dyslipidemia.

Variables	Hypertension		Diabetes		Dyslipidemia	
	AUC	95% CI	AUC	95% CI	AUC	95% CI
BF% (%)	0.600^**^	(0.589, 0.611)	0.571^**^	(0.550, 0.593)	0.600^**^	(0.590, 0.609)
BMI (kg/m^2^)	0.606^**^	**(0.595, 0.617)**	0.573^**^	**(0.552, 0.594)**	0.612^**^	(0.603, 0.621)
WHR	0.587^**^	(0.576, 0.599)	0.566^**^	(0.545, 0.588)	0.618^**^	(0.609, 0.628)
WHtR	0.606^**^	**(0.595, 0.617)**	0.573^**^	**(0.552, 0.594)**	0.625^**^	**(0.616, 0.635)**
BAI	0.582^**^	(0.571, 0.593)	0.544^**^	(0.522, 0.566)	0.576^**^	(0.567, 0.586)
OBD	0.475^**^	(0.464, 0.487)	0.491	(0.469, 0.513)	0.490^*^	(0.481, 0.500)
CI	0.573^**^	(0.561, 0.584)	0.556^**^	(0.535, 0.577)	0.615^**^	(0.606, 0.625)
AVI	0.592^**^	(0.581, 0.603)	0.568^**^	(0.547, 0.589)	0.620^**^	(0.611, 0.629)
ABSI	0.532^**^	(0.520, 0.544)	0.530^**^	(0.508, 0.552)	0.581^**^	(0.572, 0.591)
BRI	0.568^**^	(0.556, 0.579)	0.554^**^	(0.533, 0.575)	0.597^**^	(0.588, 0.607)

^*^P-value from Pearson’s correlation<0.05. ^**^P-value from Pearson’s correlation<0.001. Obesity indicators with the highest AUC values in bold.

(BF%, Body Fat Ratio; BMI, Body Mass Index; WHR, Waist-to-Hip Ratio; WHtR, Waist-to-Height Ratio; BAI, Body Adiposity Index; OBD, Obesity Degree; CI, Conicity Index; AVI, Abdominal Volume Index; ABSI, A Body Shape Index; BRI, Body Roundness Index).

## 4 Discussion

Obesity is a chronic, non-communicable condition in which body fat accumulates excessively and impairs physical and mental health, leading to lower quality of life and shorter life expectancy (29). The pathogenesis of obesity is related to genetic factors, neuropsychiatric factors, hyperinsulinemia and abnormal brown adipose tissue. Leptin is a protein encoded by the ob gene and is a hormone synthesized and secreted by fat cells (30). The changes of leptin and orexin are involved in the pathogenesis of obesity. They are two opposite polypeptides that mainly act on the hypothalamus. Leptin reduces appetite, increases energy consumption and reduces weight, while orexin stimulates eating behavior and leads to obesity. Mental factors often affect appetite, the function of the feeding center is subject to the mental state, when the mental stress is excessive and the sympathetic nerve or adrenergic nerve is stimulated (especially α receptor dominated), appetite is suppressed, while the vagus nerve excites and insulin secretion increases, appetite is often hyperactive (31). The brown adipose tissue was mainly distributed in the interscapular, neck and back, armpit, mediastinum and around the kidney, with light brown appearance and relatively small cell volume change (32). Brown adipose tissue is a thermogenic organ in function, and β3 adrenergic receptor is mainly expressed in brown fat, which is involved in the regulation of energy balance and fat storage through its thermogenic and lipolysis promoting effects. β3 adrenergic receptor gene mutation has impaired its expression in brown fat and significantly weakened its thermogenic and lipolysis promoting effects, and increased fat storage leads to obesity (33). Overeat produces too much gastric inhibitory peptide (GIP) through stimulation of the small intestine, which stimulates the release of insulin by islet beta cells. At the same time, high plasma insulin levels in obese patients can stimulate increased eating and inhibit lipolysis, causing fat accumulation in the body (34). This several factors contribute to obesity and evaluated by different obesity indicators.

This study assessed the different indicators of obesity in adults aged 35–74 years in Ningxia rural residents. In recent years, with the rapid economic development and the improvement of living standards, there have been great changes in dietary structure and lifestyle habits, physical activity and exercise have reduced gradually, and the incidence of obesity is increasing and is a serious health concern (35, 36). The prevalence of hypertension, diabetes, and dyslipidemia is increasing significantly worldwide (37–39). The prevention and treatment of obesity-related disorders is crucial, and health concerns, such as cardiovascular disease, have increased significantly. Serious cardiovascular events and related complications have affects the physical health of patients and places a heavy burden on families and society (40, 41). Therefore, it is very important to diagnose obesity earlier, and to take preventive measures to control and reduce the adverse consequences. It is necessary to develop simple, effective, and reliable screening criteria for early detection. At present, there are many indicators for diagnostic obesity, but these approaches are not uniform (42–44). This study included ten obesity indicators to explore their correlation with body measurement indicators and their cut-off values, and investigate potential relationships with hypertension, diabetes, and dyslipidemia.

Bioelectrical impedance, as a widely used method to measure human body composition, has been recognized in many medical circles (45). This study showed that the anthropometric measures were higher in men than in women, and Body Mass Index (BMI) showed no statistical significance differences between the two groups. This may be because the BMI is based on height and weight, cannot distinguish the degree of fat and muscle (46). The differences in indicators between men and women were statistically significant, suggesting that gender may be discussed in the subsequent formulation of relevant cut-off values.

Based on the correlation between obesity indicators and anthropometric and biochemical variables, waist circumference has the most influence, and some studies show that the obesity of Chinese people is mainly abdominal obesity. Related studies have pointed out that abdominal obesity is characterized by abdominal fat accumulation, which is more closely related to cardiovascular risk factors than subcutaneous adipose tissue (47). Therefore, obesity indicators should be combined with those of abdominal obesity in order to monitor the health level of the population more comprehensively. Among the various obesity indicators in this study, the calculations of Waist to hip ratio (WHR), Waist to height ratio (WHtR), Conicity index (CI), Abdominal volume index (AVI) and Body roundness index (BRI) were all related to waist circumference, among them, AVI was most significant, and also closely related to arm circumference found in this study. Studies have shown that AVI is primarily used to assess the accumulation of visceral fat, is strongly associated with impaired glucose tolerance and type 2 diabetes, and is considered a predictor of metabolic syndrome in adolescents, and suitable for community public health monitoring (48, 49). Obesity degree (OBD) did not show significant effect in this study, which may be because this indicator is mainly calculated by height, and height does not show significant influence on various obesity indicators. A large accumulation of visceral fat in obese people will produce free fatty acids and increase TG synthesis, and excessive accumulation of visceral fat will change lipase activity and increase cholesterol synthesis, leading to dyslipidemia (50). Central obesity, which is measured by waist circumference, has a greater impact on dyslipidemia (51). Studies have shown that higher ABSI indicates higher abdominal fat deposits, ABSI is closely related to visceral fat, and is positively correlated with waist circumference in both men and women (52).

Body Fat ratio (BF%) can accurately reflect the body fat content, distinguishing whether an increase in body mass is due to fat or muscle, which is considered the gold standard for evaluating obesity, and lipid molecules can affect the elastic function and structure of blood vessels, resulting in abnormal elasticity and structure of arterial blood vessels, and then cause the increase of systolic or diastolic blood pressure. Our study showed that the BF% in females was significantly higher than males, which may be related to the differences in physiological characteristics between the different sexes. Sex hormones are important factors involved in regulating fat storage, distribution, and decomposition in the body (53, 54).

Waist to height ratio WHtR had the largest AUC for predicting obesity in both gender, followed by BMI and AVI. WHtR has certain evaluation effect on hypertension and dyslipidemia in participants, which is the most suitable index for reflecting body fat distribution and central obesity, and a strong predictor of hypertension-diabetes comorbidity in adults (55). Fat accumulation in the upper body is more likely to cause diabetes and hypertension than that accumulated in the lower body. Moreover, upper body fat may directly affect fatty acids and lipid metabolism throughout the body (56, 57). Although BF% is considered the gold standard for obesity, in view of the equipment for measuring body composition is generally expensive and not suitable for handling, it is inconvenient for practical application (58). WHtR is relatively simple, and is suitable for large-scale screening of population.

This study investigated the cut-off values of different obesity indicators, which can be used as a reference for subsequent research. The use of a combination of indicators is recommended when assessing obesity, together with the fat distribution, to increase the accuracy of predicting cardiovascular disease. According to the status of each obesity diagnosis indicators, intervention to improve physical fitness and the quality of life is important (59–61).

## 5 Conclusion

The study shows that abdominal obesity is the main type of Ningxia rural adults. BMI, AVI and BRI were most affected by anthropometric indicators, especially waist circumference and arm circumference. WHtR is a suitable measure of body fat distribution and central obesity, the cutoff value is 0.52 for males and 0.55 for females. The study suggests population to pay attention to their waist circumference, to prevent cardiovascular diseases, and also could be used to monitor community health.

### 5.1 Limitations

Based on the analysis of anthropometric index data of residents in Ningxia, Northwest China, the application and cut-off value of ten obesity evaluation indicators first explored based on the body composition measurement instrument of bioelectrical impedance. This study preliminarily discussed the relationship between obesity index and related diseases, and provided basic research information for the prevention and control of obesity, hypertension, diabetes and dyslipidemia more effectively in this region and country. However, there are some limitations in this study. Firstly, part of the data collection process (such as lifestyle) is carried out by the subjects. self-reported, bias may have affected survey responses. Second, this study was only conducted on residents in rural areas of Ningxia, and due to the differences in population composition, economic level and living habits, these findings should be applied with caution to other areas. Third, population of this study only included rural residents aged 35-74. At present, we have carried out a survey of the population aged over 18 and included the urban population, stratified analysis of different indicators at different ages will be conducted to make up for the shortcomings of this study. Fourth, this study only used the baseline data of the cohort study for analysis, and the causal relationship could not be obtained. This study preliminarily discussed the relationship between obesity indicators and related diseases, and the design and analysis need to be further improved in subsequent studies.

## Data Availability

The datasets presented in this article are not readily available because The data that support the findings of this study are available from National Key Research and Development Program of China, China Northwest General Population Cohort. Restrictions apply to the availability of these data, which were used under license for this study. Data are available from the authors with the permission of project unit university. Requests to access the datasets should be directed to Yi Zhao, zhaoyi751114@hotmail.com.
